# Serum Gelsolin Combined with Albumin Might Be a Promising Marker for the Intensive Care Unit-Acquired Weakness—A Pilot Study

**DOI:** 10.3390/diagnostics16050758

**Published:** 2026-03-03

**Authors:** Zoltán Horváth-Szalai, Tihamér Molnár, Ildikó Rostás, Balázs Szirmay, Dániel Ragán, Péter Kustán, István Papp, Tamás Huber, Natália Tóth, Ákos Mérei, Attila Miseta, Tamás Kőszegi, Diána Mühl

**Affiliations:** 1 Department of Laboratory Medicine, University of Pécs Medical School, Ifjúság u. 13, 7624 Pécs, Hungary; horvath-szalai.zoltan@pte.hu (Z.H.-S.); ildiko.rostas@aok.pte.hu (I.R.); szirmay.balazs@pte.hu (B.S.); ragandaniel@hotmail.com (D.R.); peter.kustan89@gmail.com (P.K.); papp.istvan@pte.hu (I.P.); attila.miseta@aok.pte.hu (A.M.); koszegi.tamas@pte.hu (T.K.); 2 Molecular Medicine Research Group, Szentágothai Research Center, University of Pécs, Ifjúság u. 20, 7624 Pécs, Hungary; 3 Department of Anesthesiology and Intensive Therapy, University of Pécs Medical School, Ifjúság u. 13, 7624 Pécs, Hungary; toth.natalia2@pte.hu (N.T.); merei.akos@pte.hu (Á.M.); muhl.diana@pte.hu (D.M.); 4 Department of Biophysics, University of Pécs Medical School, Szigeti u. 12, 7624 Pécs, Hungary; tamas.huber@aok.pte.hu

**Keywords:** intensive care unit-acquired weakness, sepsis, gelsolin, albumin, predictive value

## Abstract

**Background/Objectives**: Intensive care unit-acquired weakness (ICUAW) is a frequent complication characterized by symmetrical and proximal limb muscle weakness. Its diagnosis is primarily based on clinical symptoms; however, ICUAW assessment can often be uncertain. Blood biomarkers have not yet been widely investigated for this purpose. Serum gelsolin (GSN) is synthesized by skeletal muscle cells. It plays a crucial role in binding extracellular actin filaments and pro-inflammatory cytokines. In sepsis-associated ICUAW, GSN levels might massively decrease due to their buffering activity and muscle wasting. We elucidated the predictive capacity of GSN regarding ICUAW and its additional diagnostic/prognostic potential in sepsis compared to classical parameters. **Methods**: We recruited septic and non-septic ICU patients for our follow-up study. Patients were retrospectively categorized into ICUAW positive (*n* = 26) and negative (*n* = 47) groups based on their clinical characteristics. Sera were collected on the 1st, 2nd and 3rd days of ICU stay. Ambulatory patients (*n* = 34) served as controls. GSN levels were measured by our previously developed automated immunoturbidimetric assay. Clinical and laboratory parameters were collected from our hospital information system. **Results**: Admission GSN levels were significantly reduced in ICU patients compared to controls (median: 11.60 vs. 75.99 mg/L). ICUAW positive patients had significantly lower admission GSN levels than ICUAW negative patients (median: 8.10 vs. 14.30 mg/L), and a similar tendency was observed during follow-up. GSN showed predictive capacity regarding ICUAW (ROC AUC: 0.711, *p* < 0.01), especially when combined with albumin (ROC AUC: 0.750, *p* < 0.01). The combination of admission GSN, albumin, and procalcitonin demonstrated significant diagnostic performance (ROC AUC: 0.803) regarding the requirement for invasive ventilation, and GSN had prognostic value for 28-day mortality as well. **Conclusions**: GSN might serve as an intriguing marker in the prediction of ICUAW, especially when combined with albumin. The parallel decline of GSN and albumin could reflect the combined effects of systemic inflammation and muscle wasting seen in ICUAW.

## 1. Introduction

Intensive care unit-acquired weakness (ICUAW) affects approximately 10–50% of critically ill patients and represents one of the most frequent and debilitating complications of prolonged intensive care treatment [1]. ICUAW is defined as clinically detected muscle weakness that develops during critical illness and cannot be explained by any alternative neurological or muscular disorder. Clinically, it is characterized by symmetrical, predominantly proximal limb weakness, with frequent involvement of respiratory muscles, including the diaphragm and intercostal muscles [2]. The development of ICUAW is associated with increased short- and long-term mortality, prolonged mechanical ventilation, extended intensive care unit (ICU) length of stay, and persistent functional impairment, resulting in a marked reduction in quality of life among survivors. Risk factors for ICUAW include old age, immobilization, mechanical ventilation, sepsis and its sequelae (e.g., multiple organ dysfunction), hyperglycemia and prolonged parenteral nutrition [2,3]. The pathophysiology of the syndrome is complex and multifactorial, involving systemic inflammation, microvascular alterations, oxidative stress, mitochondrial dysfunction, and subsequent impairment of muscle and peripheral nerve function [4]. Despite its clinical relevance, early diagnosis of ICUAW remains challenging. In awake and cooperative patients, bedside assessment of muscle strength using the Medical Research Council (MRC) sum score is considered the diagnostic gold standard for ICUAW. However, the majority of critically ill patients are sedated, delirious, or mechanically ventilated, which limits the feasibility and reliability of voluntary muscle strength testing. Electrophysiological techniques, including electromyography and nerve conduction studies, allow differentiation between critical illness myopathy (CIM), critical illness polyneuropathy (CIP), and combined forms (critical illness neuromyopathy, CINMP) [1,2,3,4]. Nevertheless, their routine application in the ICU is often constrained by technical difficulties such as limb edema, electrical interference, hemodynamic instability, and limited availability of specialized expertise. Neuromuscular ultrasound has emerged as a promising adjunctive tool for assessing muscle and nerve involvement [5]; however, it is operator-dependent, influenced by tissue edema, and lacks standardized reference values.

Given these diagnostic limitations, substantial efforts have been made to identify reliable laboratory biomarkers for ICUAW. Various inflammatory markers (e.g., C-reactive protein [CRP], procalcitonin [PCT], interleukins), skeletal muscle-derived proteins (e.g., C-terminal agrin fragment [CAF], fatty acid-binding protein 3 [FABP3], growth differentiation factor 15 [GDF15], myoglobin), markers of neuroaxonal injury, endothelial damage (syndecan-1), and cardiac injury (troponins) have been investigated, albeit with inconsistent and limited diagnostic or predictive performance [6,7,8,9,10,11,12,13,14,15,16]. To date, an early, robust, and clinically applicable serum biomarker for ICUAW is still lacking.

Gelsolin (GSN) is a multifunctional actin-binding protein present in two major isoforms in humans: a ubiquitously expressed intracellular (cytoplasmic) form (molecular weight [MW]: 80.6 kDa) and a circulating plasma/serum isoform (MW: 83 kDa), which is predominantly synthesized by skeletal muscle cells [17]. While intracellular GSN regulates actin filament dynamics, serum GSN primarily functions as an extracellular actin scavenger and anti-inflammatory buffering molecule. During physiological cell turnover, intracellular actin (G-actin; MW: 42 kDa) is released into the circulation, predominantly as filamentous actin (F-actin), and is rapidly neutralized by GSN and by another extracellular actin-binding protein, Gc (group-specific component)-globulin (MW: 52–59 kDa), which is synthesized in the liver [18,19]. In conditions associated with extensive tissue injury, such as sepsis, large amounts of actin filaments are released from necrotic cells, leading to the sequestration and depletion of circulating GSN and Gc-globulin at sites of tissue damage [20,21,22,23]. Excess free actin can act as a damage-associated molecular pattern (DAMP), promoting microthrombus formation and endothelial injury [24,25,26]. In parallel, microbial components entering the circulation during sepsis trigger a profound pro-inflammatory cytokine response. Both actin filaments and inflammatory mediators have been shown to bind serum GSN, potentially accelerating its depletion [27,28,29]. Reduced circulating levels of GSN and Gc-globulin and their diagnostic and prognostic relevance in sepsis have been demonstrated in several independent studies [20,22,23,30,31]. Beyond sepsis, decreased serum GSN concentrations have been reported in a wide range of inflammatory and tissue-damaging conditions, including polytrauma [32], acute pancreatitis [33], neurological disorders [34,35], autoimmune diseases [36], and chronic kidney disease [37].

More recently, the relationship between reduced serum GSN levels and respiratory failure has attracted increasing attention. In a multicenter observational study, GSN was identified as an independent predictor of successful weaning from mechanical ventilation in patients with acute respiratory failure [38]. In another cohort, critically low admission GSN levels in patients with community-acquired pneumonia were associated with an increased risk of ICU admission, invasive respiratory and vasopressor support, and mortality [39]. Previously, we also demonstrated that the presepsin (PSEP):GSN ratio was associated with both the invasiveness and duration of respiratory support among septic patients [40]. Collectively, these observations suggest a potential link between GSN depletion, respiratory muscle dysfunction, and the development of ICUAW.

Based on these considerations, the aim of the present pilot study was to investigate serum GSN as a potential biomarker of ICUAW and to explore its diagnostic and predictive value in critically ill patients in comparison with established clinical parameters, severity scores, and routinely used laboratory markers.

## 2. Patients and Methods

### 2.1. Studied Patients’ Groups

The study protocol was approved by the Regional Research Ethical Committee of the University of Pécs (approval number: 4327.316-2900/KK15/2011) and was conducted in accordance with the ethical principles of the Declaration of Helsinki (2013 revision). In this 3-day follow-up study, adult patients with an established diagnosis of sepsis (*n* = 65) and non-septic critically ill patients (*n* = 8) were enrolled between January 2013 and June 2019 at the Department of Anesthesiology and Intensive Care, University of Pécs, Medical School (Hungary). The present investigation was performed retrospectively through the analysis of previously collected and biobanked blood samples. Critically ill patients were categorized retrospectively according to the Sepsis-3 diagnostic criteria [41]. In addition, healthy volunteers (*n* = 34) were recruited from the outpatient clinic of the Department of Laboratory Medicine and served as controls. All participants or their legally authorized representatives received detailed oral and written information about the study protocol, and written informed consent was obtained from all prior to inclusion. Patients were assessed for withdrawal of consent or death during the study period. Critically ill patients (*n* = 73) were further stratified based on the development of intensive care unit–acquired weakness (ICUAW). ICUAW was diagnosed based on clinical findings and categorized as generalized symmetrical limb weakness and signs of respiratory muscle involvement. Accordingly, patients were classified as ICUAW-positive (*n* = 26) or ICUAW-negative (*n* = 47). Among ICUAW-negative patients, 51% were intubated at admission; however, the need for invasive mechanical ventilation was transient and rapidly resolved, while 49% required no mechanical ventilation during their ICU stay. In contrast, all ICUAW-positive patients were intubated at admission and could not be permanently weaned from mechanical ventilation due to progressive muscle weakness. To minimize the risk of misclassification, strict clinical diagnostic criteria consistent with current ICUAW definitions were applied, and patients with alternative causes of neuromuscular weakness were excluded. Specifically, none of the included patients had a history of pre-existing or newly diagnosed neuromuscular disorders, such as myasthenia gravis, Guillain–Barré syndrome, or other known myopathies or neuropathies. Sedation and medication practices that could potentially confound neuromuscular assessment were carefully considered. Deep sedation (Richmond Agitation–Sedation Scale [RASS] ≤ −2) [42] was avoided whenever possible during prolonged mechanical ventilation and was applied only transiently for specific therapeutic interventions (e.g., percutaneous tracheostomy). During the acute phase of critical illness, sedation was primarily achieved using propofol and sufentanil, with an institutional emphasis on early dose reduction and discontinuation. When feasible, patients were transitioned to dexmedetomidine, and opioid-based analgesia was replaced with non-opioid strategies. In patients undergoing major abdominal surgery, postoperative pain management was provided using epidural analgesia with bupivacaine combined with fentanyl delivered via patient-controlled analgesia (PCA).

The primary outcome of the study was to predict the development of ICUAW. Additionally, secondary analyses included the diagnostic and predictive performance of the studied markers regarding the need for invasive and non-invasive respiratory support, sepsis, septic shock and 28-day mortality.

Exclusion criteria for ICU patients included age under 18 years, presence of autoimmune disease and chronic hepatic failure. ICU patients whose weakness was deemed to be not related to the underlying critical illness were also excluded. Exclusion criteria among control patients were as follows: age under 18 years, presence of any acute inflammatory or autoimmune disease, and any form of organ insufficiency.

### 2.2. Blood Sampling and Analyses

Blood samples were taken from critically ill patients on days 1, 2, and 3 after ICU admission, when feasible. In healthy controls, a single blood sample was collected. Venous blood samples were obtained using a closed blood collection system (BD Vacutainer^®^, Franklin Lakes, NJ, USA). Samples were centrifuged after a 30 min coagulation time at 1500× *g* for 10 min. Serum aliquots were then transferred into Eppendorf tubes and stored at −80 °C until further analysis. Routine laboratory parameters, including serum albumin, creatinine, high-sensitivity C-reactive protein (hs-CRP), and procalcitonin (PCT), were measured using standard automated laboratory methods. Serum gelsolin (GSN) and Gc-globulin concentrations were determined using previously published automated immunoturbidimetric assays [23,43] in the Department of Laboratory Medicine, University of Pécs, Hungary (laboratory accreditation number: NAH-9-0008/2021).

Severity of illness was assessed on the first day of ICU admission using the Acute Physiology and Chronic Health Evaluation II (APACHE II), Simplified Acute Physiology Score II (SAPS II), and Sequential Organ Failure Assessment (SOFA) scoring systems [44,45]. Additional organ dysfunctions were evaluated based on clinical findings and laboratory parameters, according to established criteria [46,47,48]. All other clinical and laboratory data, including plasma lactate levels, mean arterial pressure (MAP), respiratory support requirements, and 28-day mortality, were extracted from the patients’ electronic medical records.

### 2.3. Statistical Analysis

Statistical analyses were performed using IBM SPSS Statistics for Windows, Version 28 (IBM Corp., Armonk, NY, USA). Data distribution was assessed using the Shapiro–Wilk test. Continuous variables were compared using the Kruskal–Wallis test or the Mann–Whitney U test, as appropriate, while categorical variables were analyzed using the chi-squared test. Correlations between continuous variables were evaluated using Spearman’s rank correlation coefficient. Associations between categorical variables were explored using relationship map analysis. Longitudinal comparisons across the follow-up period were performed using Friedman’s test, followed by post hoc Wilcoxon signed-rank tests. The diagnostic and predictive performance of the parameters investigated was assessed using receiver operating characteristic (ROC) curve analysis. Combined marker models were constructed using binary logistic regression, followed by ROC analysis. Model calibration was evaluated using the Hosmer–Lemeshow goodness-of-fit test, with *p* < 0.05 indicating acceptable model fit. Multicollinearity among independent variables was assessed using variance inflation factors (VIF), with values ≥ 5 considered indicative of significant multicollinearity; variables exceeding this threshold were excluded from further analyses. All statistical tests were two-tailed, and a *p*-value < 0.05 was considered statistically significant.

## 3. Results

### 3.1. Patients’ Characteristics: Clinical and Laboratory Parameters

Demographic, clinical and laboratory parameters of the enrolled patients are illustrated in Table 1. Approximately half (49%) of the patients belonging to the ICUAW negative group did not require invasive ventilation at admission, and 51% of them were invasively ventilated, but just for a short period (e.g., median duration of ventilation: 24 h). Patients in the ICUAW positive group were all invasively ventilated at admission, and they had repeated unsuccessful weaning trials because of progressive muscle weakness. The duration of invasive ventilation was higher (*p* < 0.001) among ICUAW positive patients than among ICUAW negative patients, and similar trends were observed regarding ICU treatment days.

The proportion of males was higher among ICU patients than that of females; however, the distribution of sex and age was similar across the two ICU patient groups. The two ICU patient groups did not differ regarding basic comorbidities. Among the critically ill patients, 32% were admitted to the ICU because of acute medical and non-surgical conditions (e.g., exacerbation of chronic obstructive pulmonary disease [COPD]; pneumonia-related acute respiratory insufficiency), while 68% were admitted after acute/extensive surgery (e.g., management of ileus, Whipple procedure). There was no significant difference in the distribution of the cause of admission between ICUAW positive and negative patients. At the time of admission, 63% of ICU patients suffered from acute kidney injury (AKI), and 24% of them required renal replacement therapy (RRT). Acute respiratory distress syndrome (ARDS) was present in 52%, acute liver dysfunction in 15%, and thrombocytopenia in 19% of the patients at admission.

All ICUAW positive patients and 83% of ICUAW negative patients suffered from sepsis. Microbiological analysis gave positive results in 80% of the septic patients, where the focus of infection varied (e.g., respiratory, abdominal, urogenital). Primary Gram-positive infection (e.g., *Staphylococcus aureus*, *Streptococcus dysgalactiae*) was present in 13.9%, Gram-negative infection (e.g., *Acinetobacter* sp., *Escherichia coli*, *Klebsiella pneumoniae*) in 12.3%, mixed (Gram-positive + negative) infection in 52.3% of the patients, while primary fungal infection (*Candida* sp.) was detected in only one patient.

The 28-day mortality rate was shown to be 45.2% among all ICU patients, which did not differ significantly between the ICUAW-negative and ICUAW-positive groups.

ICUAW-positive patients exhibited higher (*p* < 0.05) APACHE II scores than ICUAW-negative patients; however, there was no significant difference regarding SOFA and SAPS II scores. MAP values did not differ significantly between the two patients’ groups. ICU patients showed higher (*p* < 0.001) serum hs-CRP, creatinine levels, white blood cell (WBC) counts and absolute neutrophil granulocyte counts compared to controls. In accordance with these findings, ICU patients had lower (*p* < 0.01) serum albumin, Gc-globulin, hemoglobin levels and platelet counts than control individuals. Markedly increased hs-CRP and PCT levels were noted in both ICU patient groups, but without any significant difference. Similarly, there were no statistically significant differences between the two ICU patient groups in the increments of serum creatinine, plasma lactate levels, WBC counts and absolute neutrophil granulocyte counts.

### 3.2. Markers Differentiating ICUAW

**Figure 1:**
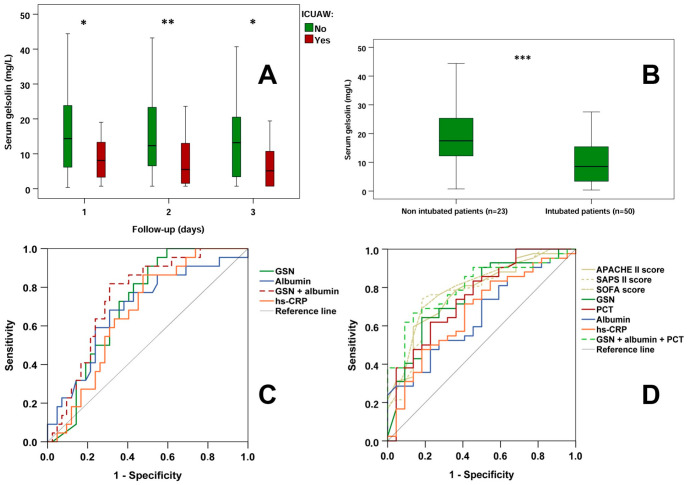
(**A**) Follow-up of serum gelsolin levels in ICUAW-negative vs. ICUAW-positive patients. (**B**) Admission serum gelsolin levels in non-intubated vs. intubated patients. (**C**) ROC analyses regarding markers predicting ICUAW; (**D**) ROC analyses indicating the requirement for invasive ventilation. ICUAW: intensive care unit-acquired weakness. *: *p* < 0.05; **: *p* < 0.01; ***: *p* < 0.001.

As highlighted in Table 1, first-day serum GSN levels were significantly reduced in both ICU patient groups compared to controls. Furthermore, ICUAW positive patients exhibited lower (*p* < 0.05) first-day serum GSN levels than ICUAW negative patients. During the follow-up, serum GSN levels remained higher in ICUAW negative patients on the 2nd (ICUAW negative vs. positive patients, median [IQR]: 12.32 [6.45–23.34] vs. 5.49 [1.32–13.45] mg/L; *p* < 0.01) as well as on the 3rd (ICUAW negative vs. positive patients: 13.18 [2.98–20.66] vs. 5.12 [1.99–11.50] mg/L; *p* < 0.05) day compared to ICUAW positive patients (Figure 1A).

Serum GSN levels were also investigated based on the type of respiratory support. At admission, 68.5% of ICU patients were invasively ventilated. First-day GSN levels were significantly lower among invasively ventilated patients than among patients without mechanical ventilation (Figure 1B).

ROC analysis revealed that for the prediction of ICUAW, 1st-day serum GSN (ROC AUC: 0.711), albumin (ROC AUC: 0.688) and hs-CRP (ROC AUC: 0.669) had significant discriminative capacity (Figure 1C, Table 2). The combined marker GSN + albumin seemed to reinforce their individual predictive performance (ROC AUC: 0.750). In this context, clinical scores (including APACHE II) did not provide any significant predictive value regarding ICUAW (Supplementary Table S1).

### 3.3. Invasive vs. Non-Invasive Respiratory Support

The diagnostic ability of the parameters studied regarding the requirement for invasive ventilation was also tested (Figure 1D, Table 2). In that case, the clinical scores APACHE II (ROC AUC: 0.772), SAPS II (ROC AUC: 0.779) and SOFA (ROC AUC: 0.797) had significant diagnostic values. Among laboratory parameters, 1st-day GSN (ROC AUC: 0.753), PCT (ROC AUC: 0.749), albumin (ROC AUC: 0.652) and hs-CRP (ROC AUC: 0.670) proved to have significant discriminatory capacity. The combined estimation of GSN, albumin, and PCT (ROC AUC: 0.803) demonstrated higher diagnostic power compared to the use of single biomarkers. Markers without any significant diagnostic capacity are listed in the Supplementary Table S1.

### 3.4. Markers and Their Diagnostic and Predictive Role for Sepsis Severity and Outcome

**Figure 2:**
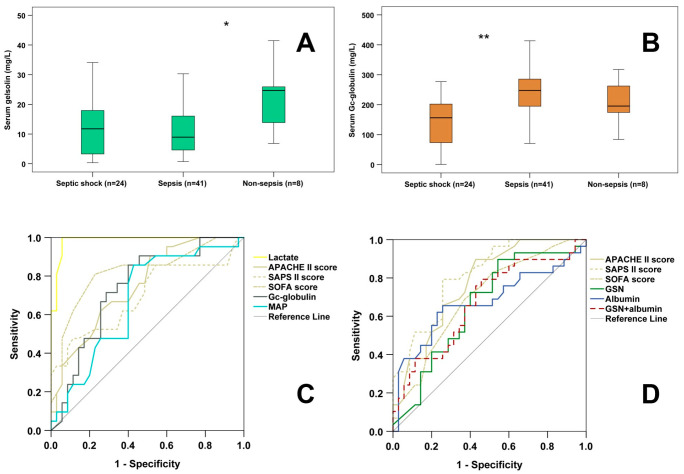
First-day serum GSN (**A**) and Gc-globulin levels (**B**) among septic shock, septic and non-septic ICU patients. ROC analyses of markers differentiating between septic shock and sepsis (**C**); and predicting 28-day mortality (**D**). *: *p* < 0.05; **: *p* < 0.01.

At admission, 56.16% of the enrolled patients were septic (without shock), 32.88% of them suffered from septic shock, while 10.96% of them were non-septic. Non-septic ICU patients showed significantly higher 1st day GSN levels than septic patients (non-sepsis vs. sepsis: 24.67 [13.11–25.95] mg/L vs. 8.95 [4.49–16.07] mg/L; *p* < 0.05) (Figure 2A). There was no significant difference in 1st-day GSN levels between septic shock and septic patients, or between septic shock and non-septic patients. When studying 1st day serum Gc-globulin concentration, septic shock patients exhibited significantly lower values compared to septic patients (septic shock vs. sepsis: 156.42 [71.68–204.75] mg/L vs. 247.62 [180.12–285.59] mg/L; *p* < 0.01) (Figure 2B). There were no significant differences when comparing septic vs. non-septic and septic shock vs. non-septic patients in terms of 1st-day serum Gc-globulin levels.

ROC analysis was not performed for the differentiation of septic vs. non-septic patients because of the limited number of non-septic individuals. Regarding the differentiation of septic vs. septic shock patients, the 1st-day clinical scores (SOFA—ROC AUC: 0.820; APACHE II—ROC AUC: 0.773; SAPS II—ROC AUC: 0.694), plasma lactate (ROC AUC: 0.985) and mean arterial pressure (MAP—ROC AUC: 0.674) demonstrated significant diagnostic performance. In addition, serum Gc-globulin also had significant discriminatory value (ROC AUC: 0.739) (Figure 2C, Table 2).

The predictive capacity of the studied markers for 28-day mortality was also tested. ROC analysis indicated that 1st-day clinical scores (SAPS II—ROC AUC: 0.814; APACHE II—ROC AUC: 0.773; SOFA—ROC AUC: 0.698), along with serum albumin (ROC AUC: 0.673) and GSN (ROC AUC: 0.659) had significant predictive values (Figure 2D, Table 2).

Markers without any significant diagnostic/predictive capacity regarding septic shock and 28-day mortality are listed in Supplementary Table S1.

### 3.5. Spearman’s Correlation Analysis for Studying the Possible Connection Between Serum Actin-Binding Proteins and Different Clinical and Laboratory Parameters

Serum GSN was positively correlated with albumin (Spearman’s correlation coefficient, ρ = 0.514, *p* < 0.001) and Gc-globulin (ρ = 0.296; *p* < 0.001) and negatively correlated with hs-CRP (ρ = −0.523; *p* < 0.001), neutrophil granulocyte count (ρ = −0.308; *p* < 0.001) and serum creatinine (ρ = −0.144; *p* < 0.05). There was an inverse association between GSN and ICU treatment days (ρ = −0.283; *p* < 0.001) and invasive ventilation days (ρ = −0.387; *p* < 0.001). Furthermore, GSN positively correlated with survival days (ρ = 0.216; *p* < 0.01).

Serum Gc-globulin was positively correlated with albumin (ρ = 0.537; *p* < 0.001) and negatively correlated with plasma lactate (ρ = −0.571; *p* < 0.001), total bilirubin (ρ = −0.375; *p* < 0.001), INR (ρ = −0.307; *p* < 0.001), neutrophil granulocyte count (ρ = −0.302; *p* < 0.001), hs-CRP (ρ = −0.238; *p* < 0.001), PCT (ρ = −0.313; *p* < 0.001) and serum creatinine (ρ = −0.248; *p* < 0.001). An inverse correlation was noted between serum Gc-globulin and APACHE II (ρ = −0.333; *p* < 0.001), SAPS II (ρ = −0.390; *p* < 0.001), and SOFA (ρ = −0.431; *p* < 0.001) clinical scores. Moreover, Gc-globulin was positively correlated with MAP (ρ = 0.317; *p* < 0.001), and survival days (ρ= 0.162; *p* < 0.05).

### 3.6. Relationship Map Analysis for Investigating Possible Associations Between Intensive Care Acquired Muscle Weakness and Different Clinical States

**Figure 3:**
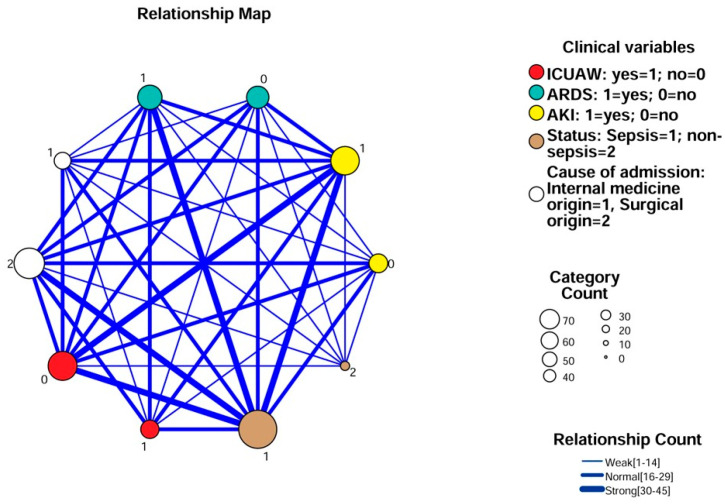
Relationship map analysis for investigating correlations between clinical variables. AKI: acute kidney injury; ARDS: acute respiratory distress syndrome; ICUAW: intensive care unit-acquired muscle weakness.

When performing relationship map analysis, possible associations were investigated between the following dichotomous clinical variables: cause of admission (internal medicine or surgical), ICUAW-positive/negative state, sepsis/non-sepsis, ARDS-positive/negative state and AKI-positive/negative state (Figure 3). The data suggested an association between the ICUAW-positive clinical state and the presence of sepsis, ARDS and AKI. The septic state itself was strongly associated with most of the clinical variables studied (admission because of surgical origin, ARDS, and AKI). However, based on the relationship map analysis, among ICUAW-negative patients, sepsis and AKI were also prevalent, demonstrating the complex interplay between different clinical variables.

## 4. Discussion

Early identification of intensive care unit-acquired weakness (ICUAW) remains a major clinical challenge. In critically ill patients, assessment of muscle strength using the Medical Research Council (MRC) sum score is frequently hampered by deep sedation, impaired consciousness, or delirium. Moreover, confounding factors such as limb edema, hemodynamic instability, or technical limitations may reduce the feasibility and reliability of electrophysiological examinations [2,3,4]. Consequently, the identification of objective laboratory biomarkers that may complement clinical evaluation and support early risk stratification is of considerable clinical interest.

Gelsolin (GSN), a protein predominantly synthesized by skeletal muscle, plays a key role in extracellular actin scavenging and immune modulation by binding actin filaments, pro-inflammatory cytokines, and microbial components, thereby functioning as an anti-inflammatory buffer [20,22,23,27,28,29]. In systemic inflammatory conditions such as sepsis, circulating GSN levels are often markedly reduced due to increased consumption during actin binding and neutralization of inflammatory mediators. In parallel, skeletal muscle wasting—one of the hallmark features of ICUAW—may further contribute to diminished GSN availability, as previously suggested [49]. Given that ICUAW develops in the context of both sustained systemic inflammation and progressive muscle degradation [2,3,4], reduced serum GSN concentrations may reflect the convergence of these pathophysiological processes.

In the present pilot study, serum GSN levels were significantly lower in ICUAW-positive patients compared with ICUAW-negative patients, and this difference was consistently observed during the first three days of ICU treatment. Moreover, lower GSN concentrations were associated with an increased likelihood of ICUAW development in exploratory predictive analyses, particularly when combined with serum albumin levels. On the first day of ICU admission, GSN concentrations were also lower in patients requiring invasive mechanical ventilation than in those without ventilatory support. In addition, the combined assessment of GSN, albumin, and procalcitonin (PCT) appeared to improve diagnostic performance for identifying patients requiring invasive respiratory support. These findings should be interpreted as hypothesis-generating and warrant confirmation in larger, prospective cohorts.

Our observations are broadly consistent with previous reports. Holm et al. [38] identified GSN as an independent predictor of successful weaning in patients with acute respiratory failure, while Self et al. [39] demonstrated that low admission GSN levels predicted the need for invasive mechanical ventilation in patients with community-acquired pneumonia. In line with these findings, our earlier work showed that the presepsin:GSN ratio differentiated mechanically ventilated septic patients from those requiring only supplemental oxygen and was associated with ventilation duration [40]. Together, these data support the concept that biomarker panels integrating reciprocal changes in inflammatory and protective mediators may offer improved prognostic value compared with single biomarkers alone.

Serum albumin, a negative acute-phase protein synthesized by the liver, exerts pleiotropic biological effects beyond maintenance of oncotic pressure, including anti-inflammatory, immunomodulatory, antioxidant, and endothelial-stabilizing functions [50]. Hypoalbuminemia is common in sepsis, largely due to systemic inflammation, capillary leakage, and altered hepatic synthesis. Notably, GSN appears to share several functional similarities with albumin, including anti-inflammatory and cytoprotective properties [27,28,29,51]. While albumin supplementation is frequently used in critically ill patients—particularly in sepsis—its definitive clinical benefit remains controversial [52]. In contrast, recombinant human GSN (rhu-GSN) therapy is currently under investigation and has shown anti-inflammatory effects in both community-acquired pneumonia and SARS-CoV-2 infection, including attenuation of pro-inflammatory cytokine production [53,54,55]. These observations raise the possibility that concomitant depletion of albumin and GSN may reflect a loss of endogenous protective capacity during critical illness, supporting the rationale for their combined assessment in exploratory prediction of ICUAW and adverse outcomes.

In our cohort, serum GSN levels were significantly lower in septic compared with non-septic ICU patients, consistent with earlier studies suggesting a diagnostic role for GSN in sepsis [22,23]. Additionally, reduced GSN concentrations were observed in non-survivors, indicating potential prognostic relevance for 28-day mortality. While these findings are concordant with several previous reports [20,22,23,56], they contrast with others [31,38], underscoring the heterogeneity of critically ill populations and the need for further validation. Alongside GSN, serum Gc-globulin—another extracellular actin-binding protein—was significantly reduced in patients with septic shock compared with those with sepsis, suggesting potential diagnostic utility in sepsis severity stratification. Correlation analyses revealed inverse associations between actin-binding proteins and inflammatory markers, as well as negative relationships between GSN and ICU length of stay and duration of invasive ventilation, and between Gc-globulin and clinical severity scores. These associations, while exploratory, further support the potential relevance of actin-binding proteins as biomarkers of disease burden in critical illness.

Finally, relationship map analysis demonstrated complex interconnections among ICUAW, sepsis, acute respiratory distress syndrome (ARDS), and acute kidney injury (AKI), indicating that ICUAW frequently coexists with other major organ dysfunctions. This observation is consistent with previous reports describing ICUAW as part of a broader syndrome of multiple organ failure rather than an isolated complication [2,3]. The strong associations between sepsis and most clinical variables examined further highlight the multifactorial nature of critical illness and the inherent difficulty of disentangling ICUAW from overlapping pathological processes.

Taken together, the findings of this single-center pilot study suggest that reduced circulating actin-binding proteins—particularly GSN, alone or in combination with albumin and inflammatory markers—may be associated with ICUAW, respiratory failure, and adverse outcomes. However, given the limited sample size, retrospective design, and exploratory nature of the analyses, these results should be interpreted cautiously. Larger, multicenter, prospective studies specifically designed to evaluate ICUAW development are required to confirm these associations and to clarify the clinical utility of actin-binding proteins as early biomarkers in critical illness.

Several limitations of the present study should be acknowledged. First, the diagnosis of ICUAW was established retrospectively and based on clinical criteria, including generalized symmetrical limb weakness and signs of respiratory muscle involvement. Electrophysiological investigations were not performed; therefore, differentiation between specific subtypes (e.g., critical illness myopathy (CIM), critical illness polyneuropathy (CIP), or combined forms (CINMP) was not possible. Although strict clinical criteria in accordance with current ICUAW definitions were applied, a certain degree of residual misclassification bias cannot be entirely excluded. To minimize this risk, patients with alternative causes of neuromuscular weakness were excluded. None of the included patients had pre-existing or newly diagnosed neurological disorders, and no patient received medications known to directly induce clinically relevant myopathy, reducing the likelihood that the observed weakness was unrelated to critical illness. Second, the study was conducted at a single center with a relatively small sample size. As a pilot investigation, it was not powered to establish definitive clinical utility, and the limited cohort increases the risk of both type I and type II errors. Consequently, the generalizability of our findings to broader ICU populations may be restricted. Third, the high prevalence of sepsis in our cohort represents a potential confounding factor. All ICUAW-positive patients were septic, and more than 80% of ICUAW-negative patients were also diagnosed with sepsis. Given that sepsis itself may reduce serum GSN levels, it cannot be fully excluded that systemic inflammation and disease severity contributed to the observed associations. Although the difference in GSN levels between ICUAW-positive and ICUAW-negative patients suggests that additional mechanisms—such as muscle wasting—may be involved, the independent contribution of ICUAW cannot be conclusively determined in this study design. Finally, the retrospective nature of the analysis and the post hoc evaluation of previously collected samples preclude causal inferences. The temporal relationship between declining GSN levels and the onset or progression of ICUAW could not be systematically assessed. Therefore, larger, multicenter, prospective studies specifically designed to evaluate ICUAW development are warranted. Such studies should incorporate standardized electrophysiological and functional assessments, predefined subgroup analyses (e.g., septic versus non-septic patients), and adequate statistical power to clarify the independent role and potential clinical utility of serum GSN in this setting.

## 5. Conclusions

Our findings suggest that serum GSN may serve as a predictor of ICUAW, especially when combined with albumin. The parallel decline of GSN and albumin, both of which have key anti-inflammatory and homeostatic roles, may predict the combined effects of systemic inflammation and muscle wasting seen in ICUAW. GSN and Gc-globulin also provide valuable insights into sepsis severity and prognosis. In the future, further studies involving a larger number of septic and non-septic patients are warranted to support these findings. Specifically, the combined assessment of serum GSN levels with clinical parameters (e.g., neuromuscular ultrasound results) may improve the early detection of ICUAW, ultimately guiding more timely and targeted interventions.

## Figures and Tables

**Table 1:** Clinical and routine laboratory parameters of the enrolled patients on admission.

Clinical and Laboratory Data	ICUAW− (*n* = 47)	ICUAW+ (*n* = 26)	Control (*n* = 34)	*p* Value
Age, years	65 (53–73)	67 (56–79)	43 (35–57)	<0.001 ^a,b^
Male, (%)	35 (74.5)	20 (76.9)	15 (44.1)	<0.05 ^a,b^
Type II. DM	11 (23.4)	5 (19.2)	-	n.s.
HT	34 (72.3)	20 (76.9)	-	n.s.
ICU treatment, days	5 (2–8)	15.5 (11–20)	-	<0.001
Invasive ventilation, days	1 (0–5)	11.5 (8–18)	-	<0.001
Non-sepsis, (%)	8 (17)	-	-	n.a.
Sepsis, (%)	39 (83)	26 (100)	-	n.s.
28-day mortality, (%)	19 (40.4)	14 (53.8)	-	n.s.
Admission parameters			-	-
APACHE II score	15 (10.2–22.7)	20 (16–25)	-	<0.05
SAPS II score	38.5 (30.3–54)	51 (29–59)	-	n.s.
SOFA score	8.5 (7–11)	10.5 (8.7–12.3)	-	n.s.
MAP, mmHg	71 (63–84)	81 (71.25–84.50)	-	n.s.
Se-creatinine, µmol/L	128 (80.8–284)	128 (64–180.5)	73 (66–86)	<0.001 ^a,b^
Se-bilirubin, µmol/L µmol/L µmol/L	10 (4.6–20.3)	11.5 (5.9–23.5)	8.2 (6.3–11.9)	n.s.
Pl-lactate, mmol/L	1.6 (0.9–2.3)	1.8 (1.3–3.1)	-	n.s.
WBC, G/L	12.9 (9.2–18)	13.1 (9.5–16.4)	6.6 (5.7–7.6)	<0.001 ^a,b^
Neu, G/L	11.2 (7.3–16.7)	10.9 (8.6–15.2)	3.7 (3–4.5)	<0.001 ^a,b^
Hemoglobin, g/L	106 (96–121)	104.5 (96.3–114.3)	146(132–153)	<0.001 ^a,b^
Thrombocytes, G/L	181 (111–265)	191 (125–257.3)	256(214–287)	<0.01 ^a,b^
Se-albumin, g/L	24.3 (20.8–28.6)	19.6 (17.1–23.7)	47 (46–49.7)	<0.001 ^a,b^
Se-hs-CRP, mg/L	207.8 (92.4–325.2)	277.9 (214.9–345.9)	1.1 (0.6–1.9)	<0.001 ^a,b^
Se-PCT, µg/L	7.2 (2.4–35.1)	14.2 (7.5–27.8)	-	n.s.
Se-GSN, mg/L	14.3 (5.5–23.9)	8.1 (3.3–13.7)	76 (68.6–84.1)	<0.05 ^a,b,c^
Se-Gc-globulin, mg/L	210.7 (142–271.6)	200 (113.5–264.9)	396 (361–415)	<0.001 ^a,b^

Data are expressed as median (%), and interquartile ranges (25–75%) are given in parentheses. Superscript lowercase letters refer to post hoc analyses: ^a^: ICUAW−–control; ^b^: ICUAW+–control; ^c^: ICUAW-–ICUAW+. APACHE II: Acute Physiology and Chronic Health Evaluation II; Gc: group-specific component; GSN: gelsolin; hs-CRP: high-sensitivity C-reactive protein; HT: hypertension; ICU: intensive care unit; ICUAW: intensive care unit-acquired weakness; MAP: mean arterial pressure; n.a.: not applicable; neu: neutrophil granulocyte; n.s.: non-significant; PCT: procalcitonin; SAPS II: Simplified Acute Physiology Score II; SOFA: Sequential Organ Failure Assessment; type II DM: type II diabetes mellitus.

**Table 2:** Detailed results of the ROC analyses for 1st-day clinical and laboratory parameters.

Differential Diagnosis	Parameter	ROC AUC (95% CI)	*p* Value
ICUAW: yes/no	GSN	0.711 (0.588–0.835)	<0.01
	Albumin	0.688 (0.550–0.827)	<0.05
	hs-CRP	0.669 (0.538–0.80)	<0.05
	GSN + albumin	0.750 (0.630–0.870)	<0.01
Intubation: yes/no	APACHE II scores	0.772 (0.652–0.891)	<0.001
	SAPS II scores	0.779 (0.656–0.901)	<0.001
	SOFA scores	0.797 (0.682–0.911)	<0.001
	GSN	0.753 (0.627–0.880)	<0.01
	PCT	0.749 (0.620–0.879)	<0.01
	Albumin	0.652 (0.515–0.788)	<0.05
	hs-CRP	0.670 (0.530–0.810)	<0.05
	GSN + albumin + PCT	0.803 (0.695–0.911)	<0.001
Sepsis vs. septic shock	Lactate	0.985 (0.960–1.0)	<0.001
	APACHE II scores	0.751 (0.624–0.878)	<0.01
	SAPS II scores	0.694 (0.540–0.847)	<0.05
	SOFA scores	0.820 (0.699–0.942)	<0.001
	Gc-globulin	0.739 (0.607–0.871)	<0.01
	MAP	0.674 (0.530–0.818)	<0.05
28-day mortality: yes/no	APACHE II scores	0.773 (0.660–0.886)	<0.001
	SAPS II scores	0.814 (0.712–0.915)	<0.001
	SOFA scores	0.698 (0.570–0.826)	<0.01
	Albumin	0.673 (0.533–0.813)	<0.05
	GSN	0.659 (0.524–0.795)	<0.05
	GSN + albumin	0.672 (0.538–0.806)	<0.05

APACHE II: Acute Physiology and Chronic Health Evaluation II; Gc: group-specific component; GSN: gelsolin; hs-CRP: high-sensitivity C-reactive protein; ICUAW: intensive care unit-acquired weakness; MAP: mean arterial pressure; PCT: procalcitonin; SAPS II: Simplified Acute Physiology Score II; SOFA: Sequential Organ Failure Assessment.

## Data Availability

The raw data supporting the conclusions of this article are available in Supplementary Material.

## Supplementary Materials

The following database can be downloaded at https://www.mdpi.com/article/10.3390/diagnostics16050758/s1: Anonymized database_GSN & ICUAW. Supplementary Table S1 is attached to the manuscript.

## Abbreviations

The following abbreviations are used in this manuscript:

AKI	Acute Kidney Injury
APACHE II	Acute Physiology and Chronic Health Evaluation II
ARDS	Acute Respiratory Distress Syndrome
GSN	Gelsolin
ICUAW	Intensive Care Unit-Acquired Weakness
SAPS II	Simplified Acute Physiology Score
SOFA	Sequential Organ Failure Assessment

## References

[1] Hermans G., Van den Berghe G. (2015). Clinical review: Intensive care unit acquired weakness. Crit. Care.

[2] Stevens R.D., Marshall S.A., Cornblath D.R., Hoke A., Needham D.M., de Jonghe B., Ali N.A., Sharshar T. (2009). A framework for diagnosing and classifying intensive care unit-acquired weakness. Crit. Care Med..

[3] Chen J., Huang M. (2024). Intensive care unit-acquired weakness: Recent insights. J. Intensive Med..

[4] Friedrich O., Reid M.B., Van den Berghe G., Vanhorebeek I., Hermans G., Rich M.M., Larsson L. (2015). The Sick and the Weak: Neuropathies/Myopathies in the Critically Ill. Physiol. Rev..

[5] Klawitter F., Walter U., Axer H., Patejdl R., Ehler J. (2023). Neuromuscular Ultrasound in Intensive Care Unit-Acquired Weakness: Current State and Future Directions. Medicina.

[6] Lei L., He L., Zou T., Qiu J., Li Y., Zhou R., Qin Y., Yin W. (2025). Predicting early diagnosis of intensive care unit-acquired weakness in septic patients using critical ultrasound and biological markers. BMC Anesthesiol..

[7] Higuchi T., Ide T., Fujino T., Tohyama T., Nagatomi Y., Nezu T., Ikeda M., Hashimoto T., Matsushima S., Shinohara K. (2025). Clinical characteristics and predictive biomarkers of intensive care unit-acquired weakness in patients with cardiogenic shock requiring mechanical circulatory support. Sci. Rep..

[8] Rosenberg B.J., Hirano M., Quinzii C.M., Colantuoni E., Needham D.M., Lederer D.J., Baldwin M.R. (2019). Growth differentiation factor-15 as a biomarker of strength and recovery in survivors of acute respiratory failure. Thorax.

[9] Bloch S.A., Lee J.Y., Syburra T., Rosendahl U., Griffiths M.J., Kemp P.R., Polkey M.I. (2015). Increased expression of GDF-15 may mediate ICU-acquired weakness by down-regulating muscle microRNAs. Thorax.

[10] Xie Y., Liu S., Zheng H., Cao L., Liu K., Li X. (2020). Utility of Plasma GDF-15 for Diagnosis and Prognosis Assessment of ICU-Acquired Weakness in Mechanically Ventilated Patients: Prospective Observational Study. BioMed Res. Int..

[11] Bloch S.A., Lee J.Y., Wort S.J., Polkey M.I., Kemp P.R., Griffiths M.J. (2013). Sustained elevation of circulating growth and differentiation factor-15 and a dynamic imbalance in mediators of muscle homeostasis are associated with the development of acute muscle wasting following cardiac surgery. Crit. Care Med..

[12] Wang B.H., Qi M.Y., Yang Z., He G.L., Meng S.Y. (2025). Growth differentiation factor-15 as a biomarker for intensive care unit-acquired weakness: A meta-analysis. Front. Med..

[13] Wang L., Long D. (2024). Correlation Between Early Serum Myoglobin Levels and the Incidence and Prognosis of Intensive Care Unit-Acquired Weakness (ICU-AW) in Septic Shock Patients: A Comparative Study. An. Acad. Bras. Cienc..

[14] Patejdl R., Walter U., Rosener S., Sauer M., Reuter D.A., Ehler J. (2019). Muscular Ultrasound, Syndecan-1 and Procalcitonin Serum Levels to Assess Intensive Care Unit-Acquired Weakness. Can. J. Neurol. Sci..

[15] Alladina J.W., Levy S.D., Hibbert K.A., Januzzi J.L., Harris R.S., Matthay M.A., Thompson B.T., Bajwa E.K. (2016). Plasma Concentrations of Soluble Suppression of Tumorigenicity-2 and Interleukin-6 Are Predictive of Successful Liberation From Mechanical Ventilation in Patients With the Acute Respiratory Distress Syndrome. Crit. Care Med..

[16] Klawitter F., Laukien F., Fischer D.C., Rahn A., Porath K., Danckert L., Bajorat R., Walter U., Patejdl R., Ehler J. (2025). Longitudinal Assessment of Blood-Based Inflammatory, Neuromuscular, and Neurovascular Biomarker Profiles in Intensive Care Unit-Acquired Weakness: A Prospective Single-Center Cohort Study. Neurocrit. Care.

[17] Feldt J., Schicht M., Garreis F., Welss J., Schneider U.W., Paulsen F. (2019). Structure, regulation and related diseases of the actin-binding protein gelsolin. Expert Rev. Mol. Med..

[18] Janmey P.A., Lind S.E. (1987). Capacity of human serum to depolymerize actin filaments. Blood.

[19] Lee W.M., Galbraith R.M. (1992). The extracellular actin-scavenger system and actin toxicity. N. Engl. J. Med..

[20] Lee P.S., Patel S.R., Christiani D.C., Bajwa E., Stossel T.P., Waxman A.B. (2008). Plasma gelsolin depletion and circulating actin in sepsis: A pilot study. PLoS ONE.

[21] Belsky J.B., Morris D.C., Bouchebl R., Filbin M.R., Bobbitt K.R., Jaehne A.K., Rivers E.P. (2016). Plasma levels of F-actin and F:G-actin ratio as potential new biomarkers in patients with septic shock. Biomarkers.

[22] Horváth-Szalai Z., Kustán P., Mühl D., Ludány A., Bugyi B., Kőszegi T. (2017). Antagonistic sepsis markers: Serum gelsolin and actin/gelsolin ratio. Clin. Biochem..

[23] Horváth-Szalai Z., Kustán P., Szirmay B., Lakatos Á., Christensen P.H., Huber T., Bugyi B., Mühl D., Ludány A., Miseta A. (2018). Predictive value of serum gelsolin and Gc globulin in sepsis—A pilot study. Clin. Chem. Lab. Med..

[24] Ahrens S., Zelenay S., Sancho D., Hanč P., Kjær S., Feest C., Fletcher G., Durkin C., Postigo A., Skehel M. (2012). F-actin is an evolutionarily conserved damage-associated molecular pattern recognized by DNGR-1, a receptor for dead cells. Immunity.

[25] Erukhimov J.A., Tang Z.L., Johnson B.A., Donahoe M.P., Razzack J.A., Gibson K.F., Lee W.M., Wasserloos K.J., Watkins S.A., Pitt B.R. (2000). Actin-containing sera from patients with adult respiratory distress syndrome are toxic to sheep pulmonary endothelial cells. Am. J. Respir. Crit. Care Med..

[26] Janmey P.A., Lamb J.A., Ezzell R.M., Hvidt S., Lind S.E. (1992). Effects of actin filaments on fibrin clot structure and lysis. Blood.

[27] Bucki R., Georges P.C., Espinassous Q., Funaki M., Pastore J.J., Chaby R., Janmey P.A. (2005). Inactivation of endotoxin by human plasma gelsolin. Biochemistry.

[28] Cheng Y., Hu X., Liu C., Chen M., Wang J., Wang M., Gao F., Han J., Zhang C., Sun D. (2017). Gelsolin Inhibits the Inflammatory Process Induced by LPS. Cell. Physiol. Biochem..

[29] Suprewicz Ł., Skłodowski K., Walewska A., Deptuła P., Sadzyńska A., Eljaszewicz A., Moniuszko M., Janmey P.A., Bucki R. (2023). Plasma Gelsolin Enhances Phagocytosis of Candida auris by Human Neutrophils through Scavenger Receptor Class B. Microbiol. Spectr..

[30] Dahl B., Schiødt F.V., Ott P., Wians F., Lee W.M., Balko J., O’Keefe G.E. (2003). Plasma concentration of Gc-globulin is associated with organ dysfunction and sepsis after injury. Crit. Care Med..

[31] Wang H., Cheng B., Chen Q., Wu S., Lv C., Xie G., Jin Y., Fang X. (2008). Time course of plasma gelsolin concentrations during severe sepsis in critically ill surgical patients. Crit. Care.

[32] Hazeldine J., Dinsdale R.J., Naumann D.N., Acharjee A., Bishop J.R.B., Lord J.M., Harrison P. (2021). Traumatic injury is associated with reduced deoxyribonuclease activity and dysregulation of the actin scavenging system. Burn. Trauma.

[33] Wollny T., Wątek M., Wnorowska U., Piktel E., Góźdź S., Kurek K., Wolak P., Król G., Żendzian-Piotrowska M., Bucki R. (2022). Hypogelsolinemia and Decrease in Blood Plasma Sphingosine-1-Phosphate in Patients Diagnosed with Severe Acute Pancreatitis. Dig. Dis. Sci..

[34] Kułakowska A., Zajkowska J.M., Ciccarelli N.J., Mroczko B., Drozdowski W., Bucki R. (2011). Depletion of plasma gelsolin in patients with tick-borne encephalitis and Lyme neuroborreliosis. Neurodegener. Dis..

[35] Pan J.W., He L.N., Xiao F., Shen J., Zhan R.Y. (2013). Plasma gelsolin levels and outcomes after aneurysmal subarachnoid hemorrhage. Crit. Care.

[36] Hu Y., Li H., Li W.H., Meng H.X., Fan Y.Z., Li W.J., Ji Y.T., Zhao H., Zhang L., Jin X.M. (2013). The value of decreased plasma gelsolin levels in patients with systemic lupus erythematosus and rheumatoid arthritis in diagnosis and disease activity evaluation. Lupus.

[37] Lee P.S., Sampath K., Karumanchi S.A., Tamez H., Bhan I., Isakova T., Gutierrez O.M., Wolf M., Chang Y., Stossel T.P. (2009). Plasma gelsolin and circulating actin correlate with hemodialysis mortality. J. Am. Soc. Nephrol..

[38] Holm F.S., Sivapalan P., Seersholm N., Itenov T.S., Christensen P.H., Jensen J.S. (2019). Acute Lung Injury in Critically Ill Patients: Actin-Scavenger Gelsolin Signals Prolonged Respiratory Failure. Shock.

[39] Self W.H., Wunderink R.G., DiNubile M.J., Stossel T.P., Levinson S.L., Williams D.J., Anderson E.J., Bramley A.M., Jain S., Edwards K.M. (2019). Low Admission Plasma Gelsolin Concentrations Identify Community-acquired Pneumonia Patients at High Risk for Severe Outcomes. Clin. Infect. Dis..

[40] Ragán D., Kustán P., Horváth-Szalai Z., Szirmay B., Miseta A., Woth G., Kőszegi T., Mühl D. (2023). Presepsin: Gelsolin ratio, as a promising marker of sepsis-related organ dysfunction: A prospective observational study. Front. Med..

[41] Singer M., Deutschman C.S., Seymour C.W., Shankar-Hari M., Annane D., Bauer M., Bellomo R., Bernard G.R., Chiche J.D., Coopersmith C.M. (2016). The Third International Consensus Definitions for Sepsis and Septic Shock (Sepsis-3). JAMA.

[42] Ely E.W., Truman B., Shintani A., Thomason J.W., Wheeler A.P., Gordon S., Francis J., Speroff T., Gautam S., Margolin R. (2003). Monitoring sedation status over time in ICU patients: Reliability and validity of the Richmond Agitation-Sedation Scale (RASS). JAMA.

[43] Horváth-Szalai Z., Kustán P., Szirmay B., Lakatos Á., Christensen P.H., Huber T., Bugyi B., Mühl D., Ludány A., Miseta A. (2018). Validation of an automated immune turbidimetric assay for serum gelsolin and its possible clinical utility in sepsis. J. Clin. Lab. Anal..

[44] Capuzzo M., Valpondi V., Sgarbi A., Bortolazzi S., Pavoni V., Gilli G., Candini G., Gritti G., Alvisi R. (2000). Validation of severity scoring systems SAPS II and APACHE II in a single-center population. Intensive Care Med..

[45] Ferreira F.L., Bota D.P., Bross A., Mélot C., Vincent J.L. (2001). Serial evaluation of the SOFA score to predict outcome in critically ill patients. JAMA.

[46] Fan E., Brodie D., Slutsky A.S. (2018). Acute Respiratory Distress Syndrome: Advances in Diagnosis and Treatment. JAMA.

[47] Khwaja A. (2012). KDIGO clinical practice guidelines for acute kidney injury. Nephron Clin. Pract..

[48] Nesseler N., Launey Y., Aninat C., Morel F., Mallédant Y., Seguin P. (2012). Clinical review: The liver in sepsis. Crit. Care.

[49] Lee P.S., Bhan I., Thadhani R. (2010). The potential role of plasma gelsolin in dialysis-related protein-energy wasting. Blood Purif..

[50] Gremese E., Bruno D., Varriano V., Perniola S., Petricca L., Ferraccioli G. (2023). Serum Albumin Levels: A Biomarker to Be Repurposed in Different Disease Settings in Clinical Practice. J. Clin. Med..

[51] Piktel E., Wnorowska U., Cieśluk M., Deptuła P., Prasad S.V., Król G., Durnaś B., Namiot A., Markiewicz K.H., Niemirowicz-Laskowska K. (2020). Recombinant Human Plasma Gelsolin Stimulates Phagocytosis while Diminishing Excessive Inflammatory Responses in Mice with *Pseudomonas aeruginosa* Sepsis. Int. J. Mol. Sci..

[52] Callum J., Skubas N.J., Bathla A., Keshavarz H., Clark E.G., Rochwerg B., Fergusson D., Arbous S., Bauer S.R., China L. (2024). Use of Intravenous Albumin: A Guideline From the International Collaboration for Transfusion Medicine Guidelines. Chest.

[53] Tannous A., Levinson S.L., Bolognese J., Opal S.M., DiNubile M.J. (2020). Safety and Pharmacokinetics of Recombinant Human Plasma Gelsolin in Patients Hospitalized for Nonsevere Community-Acquired Pneumonia. Antimicrob. Agents Chemother..

[54] Suprewicz Ł., Tran K.A., Piktel E., Fiedoruk K., Janmey P.A., Galie P.A., Bucki R. (2022). Recombinant human plasma gelsolin reverses increased permeability of the blood-brain barrier induced by the spike protein of the SARS-CoV-2 virus. J. Neuroinflamm..

[55] DiNubile M.J., Parra S., Salomó A.C., Levinson S.L. (2022). Adjunctive Recombinant Human Plasma Gelsolin for Severe Coronavirus Disease 2019 Pneumonia. Open Forum Infect. Dis..

[56] Lee P.S., Drager L.R., Stossel T.P., Moore F.D., Rogers S.O. (2006). Relationship of plasma gelsolin levels to outcomes in critically ill surgical patients. Ann. Surg..

