# Supplementary material for: Serum Gelsolin Combined with Albumin Might Be a Promising Marker for the Intensive Care Unit-Acquired Weakness—A Pilot Study
Source: Diagnostics (Basel). 2026 Mar 3;16(5):758. doi: 10.3390/diagnostics16050758 (PMC12984354; doi:10.3390/diagnostics16050758)
Supplement: Supplementary file 1 [file diagnostics-16-00758-s001.zip › diagnostics-4133426-supplementary.pdf]

Supplementary Table S1. Detailed results of the ROC analyses for 1st day clinical and laboratory parameters with no significant values.

Differential diagnosis	Parameter	ROC AUC (95% CI)	p value
ICUAW: yes/no	APACHE II scores	0.634 (0.495 – 0.773)	0.081
	SAPS II scores	0.599 (0.448 – 0.751)	0.196
	SOFA scores	0.620 (0.484 – 0.757)	0.117
	Gc-globulin	0.511 (0.360 – 0.662)	0.888
	PCT	0.613 (0.475 – 0.750)	0.142
Sepsis vs. septic shock	Albumin	0.606 (0.443 – 0.769)	0.187
	GSN	0.495 (0.331 – 0.659)	0.953
	hs-CRP	0.518 (0.355 – 0.682)	0.819
	PCT	0.654 (0.513 – 0.796)	0.055
28-day mortality: yes/no	hs-CRP	0.581 (0.440 – 0.722)	0.266
	Gc-globulin	0.588 (0.445 – 0.731)	0.227
	PCT	0.611 (0.472 – 0.749)	0.129

APACHE II: Acute Physiology and Chronic Health Evaluation II; Gc: group-specific component; GSN: gelsolin; hs-CRP: high sensitivity C-reactive protein; ICUAW: intensive care unit acquired weakness; MAP: mean arterial pressure; PCT: procalcitonin; SAPS II: Simplified Acute Physiology Score II; SOFA: Sequential Organ Failure Assessment.
